# Supplementary material for: Identification of Freezing-Responsive microRNAs and Their Targets in Chinese Jujube by Small RNA and Degradome Sequencing
Source: Int J Mol Sci. 2026 Sep 4;27(17):7912. doi: 10.3390/ijms27177912 (PMC13566890; doi:10.3390/ijms27177912)
Supplement: Supplementary file 1 [file ijms-27-07912-s001.zip › Supplementary Material S2/novel_miR_198.pdf]

Provisional ID	:	novel_miR_198
Score total	:	9286.7
Score for star read(s)	:	3.9
Score for read counts	:	9280.5
Score for mfe	:	0.7
Score for randfold	:	1.6
Score for cons. seed	:	
Total read count	:	18215
Mature read count	:	18165
Loop read count	:	0
Star read count	:	50

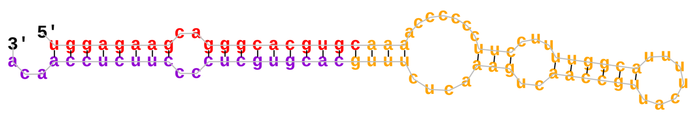

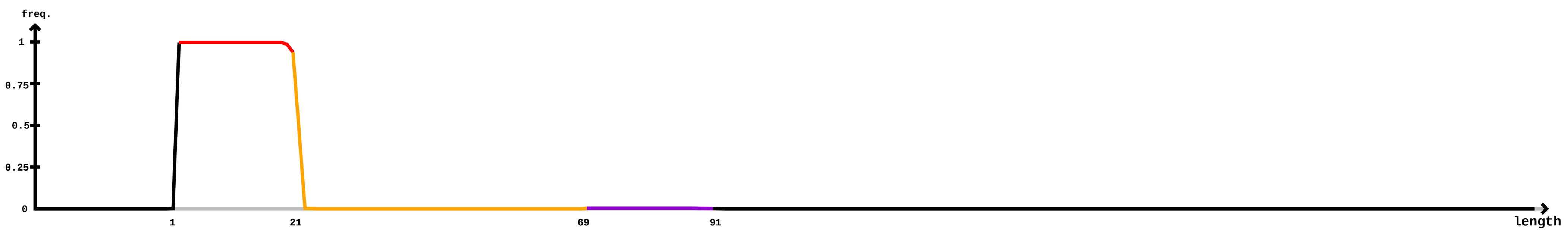

Mature
Star

```
5'- uuuuuggugugguagcuccuuguuggagaagcagggcacgugcaaaaccccccuuccuuuuggcauuuucauugccaacugaaacucuuugcacgugcuccccuucuccaacauggguuccucaccuuugucccucuauguuuaaccaccauuguauuauauauaaucuuagguaaugcaguaucaguacucuuuguuuuucuauauccucucugcuuucaguuucugauuaacauuauuuauuuauuuac  -3'   obs
    uuuuuggugugguagcuccuuguuggagaagcagggcacgugcaaaaccccccuuccuuuuggcauuuucauugccaacugaaacucuuugcacgugcuccccuucuccaacauggguuccucaccuuugucccucuauguuuaaccaccauuguauuauauauaaucuuagguaaugcaguaucaguacucuuuguuuuucuauauccucucugcuuucaguuucugauuaacauuauuuauuuauuuac        exp
    .....((((.(.(((.((.((((((((((..((((((((((((......(((...((((((......))))))..)))....))))))))))))..))))))))))).))))).).))))..........(((((........(((((((((.((......)).))))))))))(((((.(((....((.................))....)))..))))).)))))..............  reads   mm   sample
    ....................Cuggagaagcagggcacgu........................................   1       1    LT-1
    ....................uuggagaagcagggcacgugc......................................   1       0    JCK-3
    ....................uuggagaagcagggcacgugc......................................   3       0    JCK-2
    ....................uuggagaagcagggcacgugc......................................   3       0    LT-3
    ....................Cuggagaagcagggcacgugc......................................   4       1    JCK-3
    ....................Cuggagaagcagggcacgugc......................................   3       1    JCK-2
    ....................uuggagaagcagggcacgugc......................................   1       0    LT-1
    ....................Cuggagaagcagggcacgugc......................................   1       1    LT-2
    ....................Cuggagaagcagggcacgugca.....................................   1       1    LT-3
    ....................Cuggagaagcagggcacgugca.....................................   3       1    JCK-3
    ....................Cuggagaagcagggcacgugca.....................................   1       1    LT-1
    .....................uggagaagcagggcacgC........................................   1       1    LT-3
    .....................uggagaagcagggcacgu........................................   21      0    LT-2
    .....................uggagaagcCgggcacgu........................................   1       1    JCK-2
    .....................uggagaagcagggcacgu........................................   19      0    LCK-3
    .....................uggagaagcagggcacgu........................................   16      0    JCK-2
    .....................uggagaagcagggcacgu........................................   2       0    JCK-1
    .....................uggagaagcagggcacgu........................................   26      0    JT-3
    .....................uggagaagcagggcacgu........................................   34      0    LT-3
    .....................uggagaagcagggcacgu........................................   33      0    LT-1
    .....................uggagaagcagggcacgu........................................   31      0    JT-2
    .....................uggagaagcagggcacgu........................................   9       0    JCK-3
    .....................uggagCagcagggcacgug.......................................   1       1    LT-1
    .....................uggagaagcagggcacguA.......................................   7       1    LT-2
    .....................ugAagaagcagggcacgug.......................................   1       1    LT-2
    .....................uggagaagcagggcacgug.......................................   41      0    JCK-3
    .....................uggagaagcCgggcacgug.......................................   1       1    LT-2
    .....................uggagaagcagggcacgug.......................................   53      0    JCK-2
    .....................uggagaagcagggcacguC.......................................   3       1    LT-3
    .....................uggagaagcagggcacgug.......................................   66      0    LT-1
    .....................uggCgaagcagggcacgug.......................................   1       1    LT-3
    .....................uggagaagcaUggcacgug.......................................   1       1    JT-3
    .....................uggagaagcagggcacgug.......................................   122     0    LT-3
```

Mature Star

uuuuggugugguagcuccuuguuggagaagcagggcacgugcaaaaccccccuuccuuuuggcauuuucauugccaacugaaacucuuugcacgugcuccccuucuccaacauggguuccucaccuuugucccucuauguuuaaccaccauuguauuauauauaaucuuagguaaugcaguaucaguacucuuuguuuuucuauaucccucucugcuuucaguuucugauuaacauuauuuauuuauuuac

uggagaagcagggcacguA	1	1	LT-3
uggCgaagcagggcacgug	1	1	LT-2
uggagaagcagggcacgug	54	0	JT-2
uggagaagcagggcaAgug	1	1	LT-2
uggagaagcagggcacgug	76	0	JT-3
uggagaagcagggcacguA	2	1	JT-3
uggagaagcagggcacgug	122	0	LCK-2
uggagaagcagggcacgug	127	0	LCK-1
uggagaagcagggcacgug	109	0	LT-2
uggagaagcagggNacgug	1	1	LCK-1
uggagCagcagggcacgug	1	1	JT-3
uggagaagUagggcacgug	1	1	LT-1
uggagaagcagggcacgug	37	0	JCK-1
uggNgaagcagggcacgug	1	1	JCK-1
uggagaagcagggcacgug	49	0	LCK-3
uggagaagUagggcacgug	1	1	JCK-2
uggagaagcagggcacgugc	1129	0	JCK-3
uggagaagcagggcaUgugc	1	1	JCK-1
uggagCagcagggcacgugc	2	1	JCK-2
uggagaagcagggcaUgugc	2	1	JCK-2
uggagaagcagggcacgugU	25	1	LT-1
uggagaCgcagggcacgugc	2	1	LT-1
uggagaagcagggcacgugc	857	0	JT-3
uggagaagcagggNacgugc	1	1	JT-2
uggagaagcagggcacgugU	4	1	JCK-1
uggagaagcagggcacgugc	875	0	JCK-1
uggCgaagcagggcacgugc	1	1	JCK-3
uggagaagcagggcacgugc	604	0	JT-2
uggagaagcagggUacgugc	1	1	JCK-3
uggagaagUagggcacgugc	2	1	JCK-1
uAgagaagcagggcacgugc	4	1	LT-2
ugAagaagcagggcacgugc	2	1	LT-2
uggagaagcagggcacgugU	44	1	LT-2
uggagaagcagggcacgugc	1625	0	JCK-2
uggagaagUagggcacgugc	7	1	JCK-3
uggagaCgcagggcacgugc	3	1	JCK-3
uggagaagcagggcCcgugc	1	1	JT-3
uggagaagcCgggcacgugc	1	1	JT-2
uggagaagcagggcacgugU	2	1	JT-3
uggagaagUagggcacgugc	3	1	LT-2
uggagaagcGgggcacgugc	2	1	LT-3
uggagaagcagggcacgugU	11	1	JCK-3
uggagaagcagggcacgugc	318	0	LCK-2
uAgagaagcagggcacgugc	1	1	LT-1
uggaAaagcagggcacgugc	1	1	JT-3
uggagaagcagggNacgugc	1	1	LCK-1
uNgagaagcagggcacgugc	2	1	JCK-2
uggagaCgcagggcacgugc	7	1	LT-3
uggCgaagcagggcacgugc	3	1	JCK-2
uggagCagcagggcacgugc	3	1	LT-1
uggagaagcCgggcacgugc	3	1	LT-2
uggCgaagcagggcacgugc	1	1	LT-1
uggagaagcagggcacgugc	121	0	JT-1
uggagaagcagggcCcgugc	2	1	LT-2
uggagaagcagggcacgugc	1369	0	LT-2
uggagaCgcagggcacgugc	6	1	JCK-2
uggagaagcagggcCcgugc	3	1	JCK-1
uggagaagUagggcacgugc	10	1	JCK-2
uggNgaagcagggcacgugc	1	1	JCK-1
uggagaCgcagggcacgugc	3	1	LT-2
uggagaagUagggcacgugc	2	1	JT-3
ugAagaagcagggcacgugc	1	1	JCK-3
uAgagaagcagggcacgugc	1	1	JCK-1
uUgagaagcagggcacgugc	1	1	JCK-3
uggagaagcagggcacgugU	16	1	LT-3
uggagaagcagggcacgugU	21	1	JCK-2
uggagaagcagggcCcgugc	1	1	LCK-2
uggagaCgcagggcacgugc	1	1	JT-1
uggagaagcagggcacgugc	319	0	LCK-1
uNgagaagcagggcacgugc	1	1	JCK-3

Mature / **Star**

uuuuggugguguagcuccuuguuggagaagcagggcacgugcaaaaccccccuuccuuuuggcauuuucauugccaacugaaacucuuugcacgugcuccccuucuccaacauggguuccucaccuuugucccucuauguuuaaccaccauuguauuauauauaaucuuagguaaugcaguaucaguacucuuuguuuuucuauaucccucucugcuuucaguuucugauuaacauuauuuauuuauuuac

uggagaagcagggcaUgugc	2	1	LT-3
uggCgaagcagggcacgugc	11	1	LT-2
uggagCagcagggcacgugc	4	1	LT-3
uggaAaagcagggcacgugc	1	1	LT-3
Gggagaagcagggcacgugc	1	1	LCK-2
uggGgaagcagggcacgugc	1	1	LCK-3
uggagaagcagggcGcgugc	1	1	LCK-1
ugNagaagcagggcacgugc	1	1	LCK-1
uggagaagcagggcacgugc	2380	0	LT-3
Cggagaagcagggcacgugc	1	1	LT-3
uggCgaagcagggcacgugc	1	1	JCK-1
uggagaagcagggcCcgugc	1	1	JCK-3
uggagaagcagggcacgCgc	1	1	LT-3
uggagGagcagggcacgugc	1	1	LCK-1
uggagaagUagggcacgugc	1	1	LT-1
uggagaagcCgggcacgugc	5	1	LT-3
uggagaagcCgggcacgugc	1	1	LT-1
uggCgaagcagggcacgugc	4	1	LT-3
uggagaagUagggcacgugc	3	1	LT-3
uggagaagcagggcCcgugc	3	1	JCK-2
uggagaagcCgggcacgugc	1	1	JCK-3
uggagaagcCgggcacgugc	1	1	JCK-1
uggagaagcagAgcacgugc	1	1	LT-3
uggagaagcagggcacguAc	1	1	JCK-2
uggagCagcagggcacgugc	2	1	JCK-1
uggagaagcagggcacgugU	11	1	LCK-3
uggagaagcCgggcacgugc	1	1	LCK-3
uggagaagcCgggcacgugc	1	1	JCK-2
uNgagaagcagggcacgugc	1	1	LCK-1
ugAagaagcagggcacgugc	1	1	LCK-3
uggagaagcagggcacgugN	1	1	JCK-1
uggagaagcagggcCcgugc	3	1	LT-3
uNgagaagcagggcacgugc	1	1	LT-1
uggagaagcagggcacgugc	532	0	LCK-3
uggagaagcagggcNcgugc	2	1	LCK-1
uggagCagcagggcacgugc	1	1	LT-2
uggagCagcagggcacgugc	1	1	JCK-3
uAgagaagcagggcacgugc	1	1	JCK-3
Cggagaagcagggcacgugc	2	1	LT-2
uggagaagcagggcCcgugc	1	1	JT-2
Cggagaagcagggcacgugc	1	1	JCK-3
uggagaagcagNgcacgugc	2	1	JCK-1
uggagaagcagggcacgugc	2130	0	LT-1
uggCgaagcagggcacgugc	1	1	JT-2
uggNgaagcagggcacgugc	2	1	JT-2
uggagaagcaggAcacgugc	1	1	LCK-3
uggagaagAagggcacgugc	1	1	JCK-2
uggagaCgcagggcacgugc	1	1	LCK-2
ugNagaagcagggcacgugc	3	1	LT-2
uggagaagcagggcCcgugc	1	1	LT-1
uggagaCgcagggcacgugc	1	1	JT-2
uggagGagcagggcacgugc	1	1	LT-1
uggCgaagcagggcacgugc	1	1	LCK-1
uAgagaagcagggcacgugca	1	1	JCK-3
uNgagaagcagggcacgugca	5	1	JCK-2
uggagaagcagggcacgugca	131	0	JT-3
uggagaagcagggcacgugcC	4	1	LT-2
uggagaagcagggcacgugcU	19	1	LCK-3
uggagaagcagggcacgugca	360	0	LT-1
uggagaagcagggcacgugcC	2	1	JCK-1
uggagaagcagggcacgugcU	10	1	LT-2
uggCgaagcagggcacgugca	1	1	JCK-2
uggCgaagcagggcacgugca	2	1	LT-1
uggagaagcagggcacgugca	100	0	JT-1
uggagaagcagggcacgugca	2	0	LCK-2
uggagaagcagggcacgugcC	9	1	JCK-2
uggagaagcagggcacgugUa	1	1	LT-1
uggagaCgcagggcacgugca	3	1	JCK-2
uggagaagcagggcacgugcU	7	1	JCK-2
uggagCagcagggcacgugca	1	1	JCK-1

Mature **Star**

uuuuugguguguagcuccuugu**uggagaagcagggcacgugc**aaaacccccuuccuuuuggcauuuucauugccaacugaaacucuuug**cacgugcuccccuucuccaaca**uggguuccucaccuuugucccucuauguuuaaccaccauuguauuauauauaaucuuagguaaugcaguaucaguacucuuuguuuuucuauauccucucugcuuucaguuucugauuaacauuauuuauuuauuuac

uggagaagcagggcacgugcG	1	1	JCK-2
uggagCagcagggcacgugca	1	1	JCK-3
uggagaagcagggcacgugca	58	0	LCK-3
uggagaagcagggcacgugcU	6	1	LT-3
uggagaagcagggcacgugca	317	0	LT-3
uggagaagcagggcacgugNa	1	1	JCK-1
uggagaagcagggcacgugca	143	0	LT-2
uggagaCgcagggcacgugca	1	1	JCK-1
uggagaagcagggcacgugcU	16	1	JT-1
uggagaagUagggcacgugca	3	1	JCK-1
uggagaagcagggcacgugcC	7	1	LT-1
uggagaagcagggcacgugcC	3	1	LT-3
uggagaagUagggcacgugca	1	1	JT-1
uggagaagcagggcacgugca	1285	0	JCK-2
uggagaagcagNgcacgugca	2	1	JCK-1
uggagaagcagggcacgugUa	4	1	LT-2
uggagaagUagggcacgugca	1	1	LT-1
uggagaagcagggcacgugca	92	0	JT-2
uggagaagUagggcacgugca	6	1	JCK-3
uggagCagcagggcacgugca	1	1	JCK-2
uggagaagcCgggcacgugca	1	1	JCK-1
uggagaagcagggcacgugca	1008	0	JCK-3
uggagaagcagggcacgugUa	1	1	LT-3
uggagaagcagggcacgugcC	4	1	JCK-3
uggagaagcagggcaGgugca	1	1	JCK-1
uggagaagcagggUacgugca	1	1	LT-1
uggagaagcagggcaGgugca	1	1	JCK-2
uggagaagUagggcacgugca	6	1	JCK-2
uggagaagcagggcacAugca	2	1	JT-2
uggagaagcagggcaUgugca	1	1	JCK-2
uggCgaagcagggcacgugca	1	1	JCK-1
uggagaagcagggcCcgugca	1	1	LT-1
uggNgaagcagggcacgugca	1	1	JCK-1
uggagaCgcagggcacgugca	1	1	LT-3
uggagaagcagggcCcgugca	1	1	JCK-3
uNgagaagcagggcacgugca	1	1	JCK-1
uggagaagcagggcacgugcU	13	1	LT-1
uggagaagcagggcaAgugca	1	1	JCK-1
uggagCagcagggcacgugca	1	1	LT-2
uNgagaagcagggcacgugca	1	1	JCK-3
uggagaagcagggcacgugca	805	0	JCK-1
uggagaagcagggcGcgugca	1	1	JCK-3
uggagaagcagggcCcgugca	1	1	JCK-2
uggagaCgcagggcacgugca	1	1	JCK-3
uggagaagcagggcacAugca	1	1	JT-3
uggagaCgcagggcacgugca	1	1	JT-3
uggagaagcagggcacgugcU	1	1	JCK-1
uggagaagcagggcCcgugca	1	1	LT-2
uggagaagcagggcacgugcaa	3	0	JCK-3
uggagaagcagggcacgugcaa	1	0	LT-1
uggagaagcagggcacgugcaG	1	1	LT-1
uggagaagcagggcacgugcaa	3	0	JCK-2
uggagaagcagggcacgugcaa	1	0	JCK-1
uggagaagcagggcacgugcaC	1	1	JCK-1
uggagaagcagggcacgugcaaa	21	0	JT-1
uggagaagcagggcacgugcaaa	1	0	LT-1
uggagaagcagggcacgugcaCa	2	1	LT-1
gagaagcagggcacgugc	2	0	JCK-3
cacgugcuccccuucucc	1	0	JCK-1
cacgugcuccccuucucca	6	0	LCK-3
cacgugcuccccuucucca	2	0	JCK-1
cacgugcuccccuucucca	3	0	JCK-2
cacgugcuccccuucuccaa	2	0	LT-3
cacgugcuccccuucuccaac	2	0	JCK-1
cacgugcucUccuucuccaac	1	1	LT-3
cacgugcuccccuucuccaac	5	0	JCK-2
cacgugcuccccuucuccaaA	1	1	LT-1
cacgugcuccccuucuccaac	3	0	JCK-3
cacgugcuccccuucuccaac	2	0	LT-1
cacgugcuccccuucuccaacU	3	1	LT-2

```
                          Mature                                                               Star
uuuuugguguguagcuccuuguuggagaagcagggcacgugcaaaacccccccuuccuuuuggcauuuucauugccaacugaaacucuuugcacgugcuccccuucuccaacauggguuccucaccuuugucccucuauguuuaaccaccauuguauuauauauaaucuuagguaaugcaguaucaguacucuuuguuuuucuauauccucucugcuuucaguuucugauuaacauuauuuauuuauuuac
...........................................................................................cacgugcuccccuucuccaacU..........................................................................................................................................    3    1    LT-1
...........................................................................................cacgugcuccccuucuccaacU..........................................................................................................................................    5    1    JCK-1
...........................................................................................cacgugcuccccuucuccaacU..........................................................................................................................................    2    1    JCK-3
...........................................................................................cacgugcuccccuucuccaacU..........................................................................................................................................    8    1    JCK-2
............................................................................................acgugcuccccuucucca..............................................................................................................................................    1    0    JCK-2
```
